# Cost-effectiveness analysis of transarterial chemoembolization combined with lenvatinib as the first-line treatment for advanced hepatocellular carcinoma

**DOI:** 10.3389/fphar.2023.1219694

**Published:** 2023-09-07

**Authors:** Ying He, Wangchun Lin, Zhongjie Cai, Yufan Huang, Maojin You, Meisheng Lei, Ruijia Chen

**Affiliations:** ^1^ Department of Emergency Medicine, Mindong Hospital Affiliated to Fujian Medical University, Ningde, Fujian, China; ^2^ Department of Pharmacy, Mindong Hospital Affiliated to Fujian Medical University, Ningde, Fujian, China; ^3^ Department of Pharmacy, Mengchao Hepatobiliary Hospital of Fujian Medical University, Fuzhou, Fujian, China

**Keywords:** cost-effectiveness, transarterial chemoembolization, hepatocellular carcinoma, first-line treatment, TACE-LEN, lenvatinib

## Abstract

**Purpose:** Results from the LAUNCH trial suggest transarterial chemoembolization (TACE) in combination with lenvatinib is significantly more effective than lenvatinib as a first-line treatment option for advanced hepatocellular carcinoma (HCC). However, the cost of TACE is substantial. This study compares the cost-effectiveness of TACE in combination with lenvatinib (TACE-LEN) with that of lenvatinib alone as the first-line treatment for advanced HCC from the perspective of the Chinese healthcare system.

**Methods:** Markov models of different health states were constructed to simulate first-line treatment, disease progression, and survival in patients with advanced HCC. Clinical efficacy was obtained from the LAUNCH trial. The cost of drugs was sourced from national tender prices, and the treatment cost of weight-decreased was obtained from the Fujian Provincial Bureau of Prices. Other costs and utility values were based on the published literature. Total costs, life years (LYs), quality-adjusted life years (QALYs), and incremental cost-effectiveness ratios (ICERs) comprised the model output. One-way and probabilistic sensitivity analyses were performed to validate model robustness and subgroup analyses were also conducted.

**Results:** Analysis of the model showed that compared to lenvatinib, TACE-LEN improved effectiveness by 1.60 QALYs at a total cost increase of $48,874.69, with an ICER value of $30,482.13/QALY. A one-way sensitivity analysis found that the progression-free survival utility value per year had the greatest impact on the model. A probabilistic sensitivity analysis showed that TACE-LEN had a 97.9% probability of being cost-effective as the first-line treatment option for advanced HCC compared to lenvatinib when the willingness-to-pay (WTP) value was $38,201/QALY (three times the Chinese GDP *per capita* in 2022). Subgroup analysis showed that all subgroups of patients preferred TACE-LEN. However, when the WTP threshold was below $30,300/QALY, TACE-LEN is no longer cost-effective.

**Conclusion:** Our study found TACE-LEN to be a cost-effective treatment option for patients with advanced HCC compared to lenvatinib from a Chinese healthcare system perspective, but not so in low-income provinces in China.

## 1 Introduction

Primary liver cancer is one of the most frequent malignant tumors in the world, ranking as the sixth most common cancer and the third leading cause of cancer-related deaths. Approximately 906,000 new diagnoses and 830,000 deaths occurred due to liver cancer in 2020 alone (Sung et al., 2021). In China, the incidence and mortality rate of primary liver cancer ranks fourth and second, respectively, in the category of malignancies (Chen et al., 2016). Hepatocellular carcinoma (HCC) is the most common type of liver cancer, accounting for approximately 90% of cases (Llovet et al., 2021). China is also one of the high-risk regions for HCC (Sung et al., 2021). Most patients are diagnosed with HCC which has progressed to an advanced stage and is no longer amenable to radical treatments such as surgery (Forner et al., 2018). Lenvatinib, an oral tyrosine kinase inhibitor, is recommended as the standard first-line treatment for advanced HCC (Chen et al., 2020). Unfortunately, the efficacy of lenvatinib is unsatisfactory, with a median overall survival (OS) of only 13.6 months when administered as the first-line treatment for advanced HCC (Kudo et al., 2018).

Transarterial chemoembolization (TACE) is majorly used for the palliative treatment of patients with advanced HCC (Wu et al., 2017). However, a considerable number of patients are insensitive or resistant to TACE alone (Kudo et al., 2014), probably due to the upregulation of vascular endothelial growth factor (VEGF) and fibroblast growth factor (FGF) after TACE is performed (Sergio et al., 2008). Lenvatinib is an anti-angiogenic drug that can inhibit VEGF and FGF, thus inhibiting tumor angiogenesis and tumor cell proliferation (Kudo, 2018). In addition, TACE may improve the antitumor activity of lenvatinib by reducing the tumor load (Lencioni et al., 2016; Peng et al., 2022). Therefore, the synergistic anti-tumor properties of TACE and lenvatinib appear promising. A recent study in China (LAUNCH trial) evaluated the efficacy and safety of TACE in combination with lenvatinib (TACE-LEN) for the treatment of advanced HCC (Peng et al., 2022). TACE-LEN significantly prolonged median overall survival (OS) (17.8 vs. 11.5 months) and median progression-free survival (PFS) (10.6 vs. 6.4 months) in patients with advanced HCC compared to lenvatinib and was associated with only mild adverse effects (Peng et al., 2022). Thus, the findings of the LAUNCH trial bring hope to patients with advanced HCC, but the high cost of TACE also carries a heavy economic burden on patients and the national healthcare system. To the best of our knowledge, there are presently no economic evaluations of TACE-LEN for advanced HCC. In our study, we used Markov models to perform a pharmacoeconomic evaluation of the two treatment strategies (TACE-LEN vs. lenvatinib) for the treatment of advanced HCC, from a Chinese healthcare system perspective.

## 2 Materials and methods

The study was designed following the Consolidated Health Economic Evaluation Reporting Standards (CHEERS) reporting guidelines (Supplementary Table SA) (Husereau et al., 2022).

### 2.1 Model structure

**FIGURE 1:**
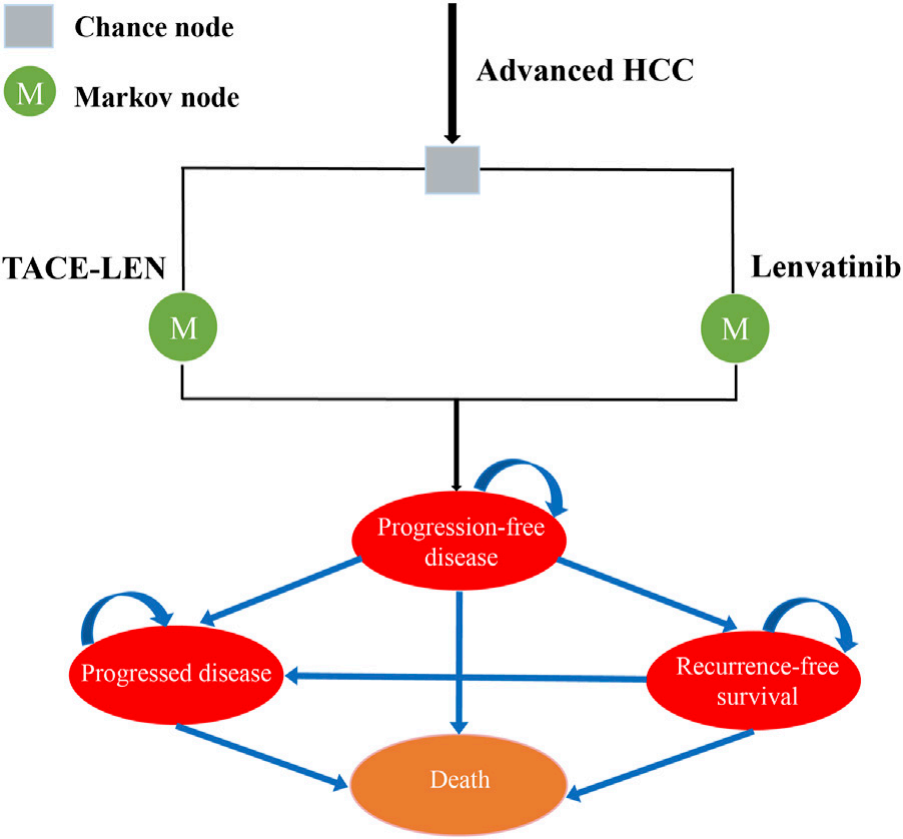
Markov model simulating outcomes for the LAUNCH trial. All patients with advanced HCC started with PFS state and received treatment with TACE-LEN or lenvatinib. HCC, hepatocellular carcinoma; TACE-LEN, transarterial chemoembolization in combination with Lenvatinib.

A Markov model was developed using TreeAge Pro 2022 (TreeAge Software, Williams-town, MA) to compare the cost-effectiveness of two regimens (TACE-LEN vs. lenvatinib) as the first-line treatment for advanced HCC. The model included four different health states: PFS, recurrence-free survival (RFS), progressive disease (PD), and death. All the health states were mutually exclusive (Figure 1). All patients were in the PFS state at the start of treatment, and as treatment progressed, patients were allowed to remain in their current health state or move to the next health state. Patients were not allowed to return to their previous healthy state. The time horizon of the model was approximately 11 years (determined as the time point at which 99% of the patients in the cohort died), with each cycle in the model being 21 days. Our cost-effectiveness analysis was conducted from the perspective of the Chinese healthcare system. The model output included total cost, life years (LYs), quality-adjusted life years (QALYs), and incremental cost-effectiveness ratios (ICERs). We set the willingness-to-pay (WTP) threshold to $38,201/QALY (three times the GDP *per capita* in China in 2022), as recommended by the World Health Organization (Cameron et al., 2018; Ochalek et al., 2020). If the ICER value was lower than the predefined WTP threshold, we then considered TACE-LEN to be cost-effective compared to lenvatinib as the first-line regimen for advanced HCC.

### 2.2 Clinical data

Patients included were consistent with the population characteristics of the LAUNCH trial (Peng et al., 2022), a randomized phase III clinical trial conducted in 12 hospitals in China from June 2019 to July 2021 with the following criteria: 1) age 18–75 years; 2) advanced primary HCC without any previous treatment or advanced HCC that has not received any postoperative treatment after hepatectomy and has recurred for the first time; 3) at mRECIST19 basis, with at least one measurable lesion in the liver; intrahepatic lesions consisting of a single tumor or multiple tumors with 50% tumor burden; 4) Eastern Cooperative Oncology Group performance status score of 0 or 1; 5) Child-Pugh class A; 6) life expectancy of 3 months or more. These patients were randomly assigned to receive either TACE-LEN or lenvatinib. To simplify the model, we assumed that all patients took 12 mg of lenvatinib daily and were discontinued when disease progression or unacceptable toxicity occurred. Patients in the TACE-LEN group started TACE treatment 1 day after oral lenvatinib and underwent TACE again if incomplete necrosis and tumor regeneration were detected. TACE was discontinued if disease progression occurred or it could not be administered. Economic analyses were based on published randomized clinical trials and mathematical models. As a result, institutional review board or ethics committee approval was not necessary for this study.

### 2.3 Transition probabilities

**FIGURE 2:**
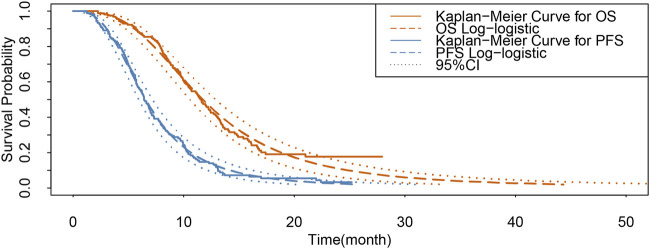
Results of the survival curve fit the lenvatinib group. 95% CI: 95% confidence interval; OS, overall survival; PFS, progression-free survival.

The probabilities of PFS and OS in Kaplan–Meier survival curves of patients in the lenvatinib group from the LAUNCH trial (Peng et al., 2022) were extracted by GetData Graph Digitizer (version 2.26) (Wan et al., 2019). Individual patient data for each Kaplan–Meier curve were reconstructed and the data were fitted using R software (version 4.2.0) using survival extrapolation to obtain long-term clinical survival functions, according to the method described by Hoyle and Henley (2011). The best-fit survival functions were selected based on the Akaike information criterion (AIC) and Bayesian information criterion (BIC) tests, in that lower AIC and BIC values indicated a better fit (Williams et al., 2017). The AIC and BIC values for each type of survival distribution function for PFS and OS curves are shown in Supplementary Table SB; Supplementary Figure SA. Also, external validation of our extrapolated survival function was performed using the results of Kudo et al. (2018a) according to the method of Latimer (2013). Ultimately, the log-logistic distribution function [S(t)= (1+(λt)^γ^)^−1^; S: survival probability, t: time cycle, λ: scale parameter, and γ: shape parameter] provided the best fit for the PFS and OS data of the patients in the lenvatinib group and was used to generate the probability of transition for the lenvatinib strategy (Table 1; Figure 2). The PFS and OS data for the TACE-LEN group were calculated based on the hazard ratio (HR) for the TACE-LEN group versus that for the lenvatinib group as reported in the LAUNCH trial (Peng et al., 2022). In the LAUNCH trial (Peng et al., 2022), after the institution of the first-line treatment, 15.3% and 1.8% of patients in the TACE-LEN and lenvatinib groups, respectively, underwent hepatectomy due to down-staging, and these patients subsequently entered RFS status, while those with recurred after hepatectomy entered PD status. Because the LAUNCH trial (Peng et al., 2022) did not provide data on the risk of recurrence of HCC after hepatectomy, we assumed a 5-year recurrence rate of 19% after hepatectomy in the model as reported by Zheng et al. (2017). Meanwhile, the 7-year (20%) and 3-year (15%) recurrence rates were used as the upper and lower bounds for sensitivity analysis, respectively (Zheng et al., 2017; Li et al., 2021; Zhang et al., 2022). We assumed that the transition probability from the PFS state to the death state is the natural mortality rate of the Chinese population in 2022 (7.4‰) (Compiled by National Bureau of Statistics of China, 2023). All patients received the best supportive care (BSC) after disease progression, including aggressive analgesia, correction of hypoalbuminemia, intensive nutritional support, and management of complications such as ascites, jaundice, and hepatic encephalopathy (The General Office Of The National Health and Health Commission, 2022).

**TABLE 1:** The basic parameters of the input model and the range of sensitivity analyses.

Variable	Base Value	Range		Distribution	Source
		Min	Max		
Log-logistic survival model of PFS for lenvatinib group					
Scale (λ)	0.1524227	0.121938	0.182907	Log-logistic	Peng et al. (2022)
Shape (γ)	2.850079	2.2800632	3.4200948	Log-logistic	Peng et al. (2022)
Log-logistic survival model of OS for lenvatinib group					
Scale (λ)	0.08526536	0.068212	0.102318	Log-logistic	Peng et al. (2022)
Shape (γ)	2.926645	2.341316	3.511974	Log-logistic	Peng et al. (2022)
HR of TACE-LEN group versus lenvatinib group					
HR for PFS	0.43	0.34	0.60	Log-normal	Peng et al. (2022)
HR for OS	0.45	0.33	0.60	Log-normal	Peng et al. (2022)
TACE-LEN group: incidence of AEs					
Hyperbilirubinemia	0.094	0.075	0.113	Beta	Peng et al. (2022)
Elevated ALT/AST	0.406	0.325	0.487	Beta	Peng et al. (2022)
Weight decreased	0.076	0.061	0.091	Beta	Peng et al. (2022)
Hypertension	0.206	0.165	0.247	Beta	Peng et al. (2022)
Diarrhea	0.053	0.042	0.064	Beta	Peng et al. (2022)
Lenvatinib group: incidence of AEs					
Hyperbilirubinemia	0.030	0.024	0.036	Beta	Peng et al. (2022)
Elevated ALT/AST	0.030	0.024	0.036	Beta	Peng et al. (2022)
Weight decreased	0.071	0.057	0.085	Beta	Peng et al. (2022)
Hypertension	0.196	0.157	0.235	Beta	Peng et al. (2022)
Diarrhea	0.042	0.034	0.050	Beta	Peng et al. (2022)
Cost ($)					
Hyperbilirubinemia	124.90	99.92	149.88	Gamma	Wen et al. (2021)
Elevated ALT/AST	45.60	36.48	54.72	Gamma	Li et al. (2021)
Weight-decreased	75.20	60.16	90.24	Gamma	Local charge
Hypertension	1.48	1.18	1.78	Gamma	Wen et al. (2021)
Diarrhea	3.61	2.89	4.33	Gamma	Wen et al. (2021)
Hepatectomy	9058.24	7246.59	10869.89	Gamma	Zhang et al. (2022)
Hospitalization per cycle	384.00	307.20	460.80	Gamma	Zhang et al. (2022)
TACE per cycle	1929.00	1543.20	2314.80	Gamma	Zhang et al. (2022)
BSC per cycle	363.00	290.40	435.60	Gamma	Zhang et al. (2022)
Test per cycle	359.96	287.97	431.95	Gamma	Li et al. (2021)
End-of-life care	2176.00	1740.80	2611.20	Gamma	Kang et al. (2021)
Lenvatinib	1054.88	843.90	1265.86	Gamma	Yao (2023)
Utility value					
PFS	0.76	0.608	0.912	Beta	Li et al. (2021)
PD	0.68	0.544	0.816	Beta	Li et al. (2021)
RFS	0.76	0.608	0.912	Beta	Li et al. (2021)
Discount rate (%)	5.00	0.00	8.00	Fixed	Liu et al. (2011)
Proportion					
Undergoing hepatectomy after TACE-LEN	0.153	0.122	0.184	Beta	Peng et al. (2022)
Undergoing hepatectomy after lenvatinib	0.018	0.014	0.022	Beta	Peng et al. (2022)
Recurrence of HCC	0.19	0.15	0.20	Beta	Zheng et al. (2017), Zhang et al. (2022)

AE, adverse event; ALT, alanine transaminase; AST, aspartate transaminase; BSC, best supportive care; HCC, hepatocellular carcinoma; HR, hazard ratio; OS, overall survival; PD, progression of disease; PFS, progression-free survival; RFS, recurrence-free survival; TACE, transarterial chemoembolization; TACE-LEN, transarterial chemoembolization in combination with Lenvatinib.

### 2.4 Costs and health utility values

We considered only direct medical costs in our model, including the costs of drugs, hospitalization, tests, hepatectomy, end-of-life care, management of adverse reactions with an incidence greater than 5%, and BSC (Table 1). Based on the LAUNCH trial (Peng et al., 2022), patients in the TACE-LEN group received a mean of three TACE treatments, and patients in the lenvatinib group received lenvatinib for an average duration of 5.1 months (approximately 7 cycles). The treatment cost of weight decreased adverse reaction was taken from the Fujian Provincial Price Bureau, and the cost of drugs was from the national tender price. Other costs were sourced from published literature and adjusted to 2022 values using the China Statistics Bureau Medical Price Index (Compiled by National Bureau of Statistics of China, 2023). All costs are expressed in US dollars, converted at the average exchange rate in 2022 ($1 = 6.73 RMB). Health-related quality of life was extracted to calculate cost-effectiveness in each group. Since quality of life was not assessed in the LAUNCH trial, we obtained the utility values (EQ-5D) for PFS, RFS, and PD from the National Institute for Health and Clinical Excellence (NICE) technology appraisal guidance 189 (NICE, 2017) and published literature (Cammà et al., 2013; Qin et al., 2018; Li et al., 2021; Zhang et al., 2022). Both costs and utility values were discounted, and the discounted value was set at 5% per year (Liu G et al., 2011).

### 2.5 Model results and sensitivity analysis

Total cost, LYs, QALYs, and ICERs constituted the model output. To identify the variables that have the greatest influence on the model outputs, we conducted a one-way sensitivity analysis, the results were represented as a tornado diagram, we let the value of each variable in the model fluctuate at a certain level, and the fluctuation range was derived from published literature. The variation range used ±20% of the baseline value in the absence of data. The lower and upper values of the discount rate were set at 0% and 8%, respectively (Liu G et al., 2011). In addition, to verify the influence of the parameters on the uncertainty of the model, we performed a probabilistic sensitivity analysis with Monte Carlo simulations of the model with 1,000 replications. To this end, specific distributions of the parameters were chosen as appropriate, as shown in Table 1. The results of the probabilistic sensitivity analysis are represented by cost-effectiveness acceptability curves and scatter plots. At the same time, we explore the changes in the TACE-LEN cost-effectiveness probability by continuously reducing the WTP threshold to meet the needs of Chinese provinces, which differ significantly from each other in terms of their economic development levels.

### 2.6 Subgroup analysis

We performed a subgroup analysis of all patients using the method prescribed by Hoyle et al. (2010) using specific HRs of subgroups reported in the LAUNCH trial (Table 2) (Peng et al., 2022).

**TABLE 2:** Results for subgroup analyses.

Subgroup	PFS HR (95% CI)	OS HR (95% CI)	ICER ($/QALY)	Cost-effectiveness probability (%)
Age, years				
60 and younger	0.37 (0.27–0.50)	0.42 (0.29–0.61)	31,234.18	96.7
older than 60	0.55 (0.35–0.84)	0.52 (0.30–0.89)	29,046.99	98.0
Sex				
Male	0.43 (0.33–0.56)	0.43 (0.31–0.60)	30,422.54	97.4
Female	0.46 (0.27–0.80)	0.52 (0.27–1.00)	30,085.37	96.0
Bodyweight, kg				
<60	0.42 (0.28–0.64)	0.46 (0.28–0.76)	30,582.50	96.4
≥60	0.44 (0.32–0.59)	0.43 (0.30–0.63)	30,277.62	96.6
Aetiology				
HBV	0.43 (0.33–0.56)	0.47 (0.34–0.64)	30,523.71	97.1
Others	0.44 (0.22–0.89)	0.34 (0.15–0.78)	29,822.47	97.2
ECOG–PS				
0	0.46 (0.33–0.64)	0.40 (0.26–0.62)	29,946.08	98.7
1	0.33 (0.22–0.48)	0.45 (0.29–0.70)	31,945.73	93.3
AFP, ng/mL				
<400	0.53 (0.38–0.75)	0.50 (0.33–0.77)	29,304.15	98.4
≥400	0.35 (0.25–0.51)	0.39 (0.26–0.61)	31,421.92	93.9
ALBI grade				
Grade 1	0.36 (0.22–0.58)	0.47 (0.27–0.82)	31,471.43	93.0
Grade 2	0.46 (0.34–0.61)	0.44 (0.31–0.63)	30,039.09	98.1
No. of tumor				
Single	0.44 (0.25–0.76)	0.55 (0.27–1.11)	30,365.88	94.0
Multiple	0.44 (0.34–0.58)	0.43 (0.31–0.60)	30,278.26	97.2
Main tumor size, cm				
<5	0.46 (0.29–0.73)	0.38 (0.20–0.71)	29,848.91	97.2
≥5	0.42 (0.32–0.56)	0.47 (0.33–0.66)	30,658.87	95.2
Primary tumor				
Yes	0.44 (0.34–0.57)	0.44 (0.32–0.61)	30,318.34	97.3
No	0.37 (0.16–0.86)	0.30 (0.07–1.34)	30,690.05	96.3
PVTT				
Yes	0.31 (0.23–0.41)	0.34 (0.24–0.49)	31,880.65	93.1
No	0.67 (0.43–1.05)	0.72 (0.40–1.29)	27,746.96	98.5
EHS				
Yes	0.46 (0.33–0.63)	0.56 (0.38–0.82)	30,274.72	97.2
No	0.40 (0.28–0.59)	0.32 (0.19–0.52)	30,426.26	97.4

AFP, a-fetoprotein; ALBI, albumin-bilirubin score; ECOG-PS, eastern cooperative oncology group performance status; EHS, extrahepatic spread; HBV, hepatitis B virus; HR, hazard ratio; ICER, incremental cost-effectiveness ratio; OS, overall survival; PFS, progression-free survival; PVTT, portal vein tumor thrombus; QALY, quality-adjusted life years; TACE, transarterial chemoembolization.

### 2.7 Scenario analysis

We analyzed five different scenarios across the overall population. Firstly, we set different 5-year recurrence probabilities after HCC surgery (15%, 20%) to assess the impact of postoperative recurrence rates on the model outcomes. Secondly, the model’s time horizon was varied to 3 years, 5 years, and 7 years to evaluate its robustness as much as possible. Thirdly, we assumed that only 80% or 50% of patients received BSC after disease progression, simulating some patients in clinical practice who discontinue treatment due to certain reasons. Fourth, the daily dosage of lenvatinib for all patients has been changed to 8 mg or 10 mg. Fifth, in the base case analysis, we made the conservative assumption that the probability of a patient dying directly from PFS status was assumed to be equal to the natural mortality rate in the Chinese population. To assess the impact of this assumption on the model results, we conducted a scenario 5 analysis. In this scenario, we adjusted the probability that a patient with PFS state would die outright by setting it at 2 or 4 times the natural mortality rate of the Chinese population.

## 3 Results

### 3.1 Base case analysis

The results of the cost-effectiveness analysis of the model are shown in Table 3. The lenvatinib group obtained 1.57 LYs and 1.05 QALYs at a total cost of $37,379.93, while the TACE-LEN group obtained 4.17 LYs and 2.65 QALYs at a total cost of $51,333.21. Compared to the lenvatinib group, the TACE-LEN group had an ICER value of $30,482.13/QALY, which was lower than the predetermined WTP value ($38,201/QALY). In other words, compared to Lenvatinib, TACE-LEN was found to be a cost-effective treatment option as the first-line regimen for advanced HCC.

**TABLE 3:** Main results of the model output.

Regimen	TACE-LEN	Lenvatinib	Incremental
Overall cost ($)	86,254.63	37,379.93	48,874.69
Overall LYs	4.17	1.57	2.60
Total QALYs	2.65	1.05	1.60
ICER, ($)			
per LY			18,800.48
per QALY			30,482.13

ICER, incremental cost-effectiveness ratio; LY, life year; QALY, quality-adjusted life year; TACE-LEN, transarterial chemoembolization in combination with Lenvatinib.

### 3.2 Sensitivity analysis

**FIGURE 3:**
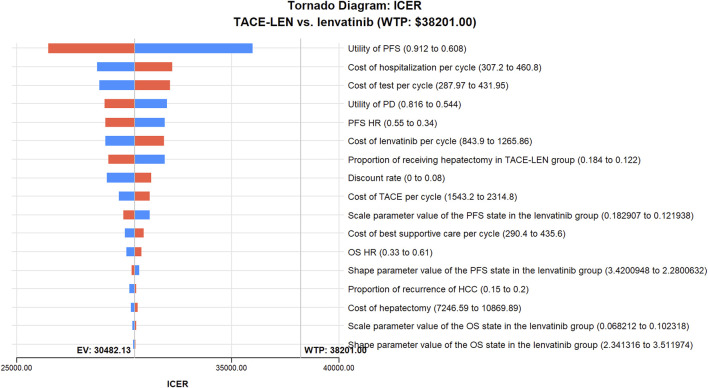
One-way sensitivity analyses of TACE-LEN in comparison with lenvatinib. HCC, hepatocellular carcinoma; HR, hazard ratio; ICER, incremental cost-effectiveness ratio; OS, overall survival; PD, progression of disease; PFS, progression-free survival; QALY, quality-adjusted life year; TACE, transarterial chemoembolization; TACE-LEN, transarterial chemoembolization in combination with lenvatinib; WTP, willingness-to-pay.

**FIGURE 4:**
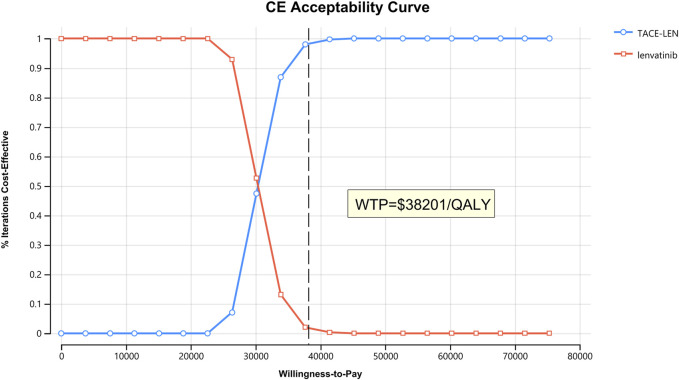
The cost-effectiveness acceptability curves for the TACE-LEN treatment option compared with the lenvatinib treatment option. QALY, quality-adjusted life year; TACE-LEN, transarterial chemoembolization in combination with lenvatinib; WTP, willingness-to-pay.

As per the results of the one-way sensitivity analysis (Figure 3), parameters with the greatest impact on the model results included the PFS utility value per year, the proportion of the TACE-LEN group undergoing hepatectomy, and the discount rate per year. Meanwhile, parameters with a lesser impact on the model results included the cost of the test per cycle, the cost of lenvatinib per cycle, and the cost of hospitalization per cycle. Although these parameters had some impact on the model results, the ICER was consistently lower than the predetermined WTP value ($38,201/QALY) when these parameters were varied within a predetermined range. The results of the probabilistic sensitivity analysis are shown in Figure 4; Supplementary Figure SB. The cost-effectiveness acceptability curve shows that the probability of cost-effectiveness of TACE-LEN increased as the WTP threshold increased. Moreover, when the WTP threshold reached our pre-set threshold ($38,201/QALY), the probability of TACE-LEN being cost-effective as the first-line regimen for HCC was 97.9%.

### 3.3 Subgroup analysis

The results of the subgroup analysis showed that TACE-LEN was cost-effective at a WTP threshold of $38,201/QALY when compared to lenvatinib as a first-line treatment option for advanced HCC, regardless of the baseline characteristics of the patients (Table 2). This further validates TACE-LEN was a cost-effective first-line treatment option for advanced HCC.

### 3.4 Scenario analysis

In scenario 1, we found that the change in recurrence rate after HCC had little effect on the ICER. In scenario 2, the model’s time horizon changes to 3, 5, and 7 years, and the ICERs are $37,978.51/QALY, $34,337.91/QALY, and $32,549.85/QALY, respectively, which shows that as the model runs longer, the ICER value decreases, meaning that the LEN-TACE regimen is more cost-effective. In scenario 3, the ICERs for LEN-TACE versus lenvatinib were $30,041.191/QALY and $29,379.78/QALY, respectively, when 80% or 50% of patients received BSC. In scenario 4, when patients took lenvatinib at a dose of 8 mg or 10 mg per day, the ICERs for TACE-LEN compared to lenvatinib were $29702.731/QALY and $31094.91/QALY, respectively. In scenario 5, when the probability of a patient dying directly from PFS state was 14.8‰ or 29.6‰ per year, the ICERs for TACE-LEN compared to lenvatinib were $29,498.16/QALY and $29,817.47/QALY, respectively. The results of the scenario analysis are shown in Table 4.

**TABLE 4:** Results for scenario analyses of the overall population.

Scenarios	Cost ($)		QALY		ICER ($/QALY)
	TACE-LEN	Lenvatinib	TACE-LEN	Lenvatinib	
Scenario 1					
Recurrence of HCC = 0.15	86,581.33	37,405.30	2.68	1.05	30,224.53
Recurrence of HCC = 0.20	86,172.75	37,373.56	2.65	1.05	30,547.07
Scenario 2					
Model runtime (year) = 3	64,869.54	34,819.84	1.73	0.94	37,978.51
Model runtime (year) = 5	75,380.32	36,299.27	2.14	1.00	34,337.91
Model runtime (year) = 7	80,256.90	36,831.81	2.36	1.03	32,549.85
Scenario 3					
80% of patients receive BSC	84,560.74	36,393.04	2.65	1.05	30,041.19
50% of patients receive BSC	82,019.91	34,912.71	2.65	1.05	29,379.78
Scenario 4					
Receive a daily dose of lenvatinib (mg) = 8	78,853.94	33,647.44	2.65	1.05	29,702.73
Receive a daily dose of lenvatinib (mg) = 10	82,549.84	35,511.45	2.65	1.05	31,094.91
Scenario 5					
Probability of direct death in patients with PFS state = 14.8‰	81,541.35	35,334.80	2.61	1.04	29,498.16
Probability of direct death in patients with PFS state = 29.6‰	79,615.02	34,990.08	2.53	1.03	29,817.47

BSC, best supportive care; HCC, hepatocellular carcinoma; ICER, incremental cost-effectiveness ratio; TACE-LEN, transarterial chemoembolization in combination with Lenvatinib; QALY, quality-adjusted life year.

## 4 Discussion

For many years, although there are many treatment options for HCC, such as sorafenib and lenvatinib, the true clinical benefit obtained from these therapeutic regimens has been less than satisfactory, and researchers have been working on exploring new drugs or treatment modalities (Ho et al., 2018; Peng et al., 2022). TACE is the basic treatment for mid to late-stage HCC, and its short-term efficacy is very good, but its long-term efficacy is not satisfactory (Palmer et al., 2020). The emergence of targeted and immunotherapy has enriched the treatment of liver cancer, and the addition of targeted and immunotherapy to TACE can allow patients to achieve longer-term survival. In the choice of a combination therapy regimen, TACE combined with targeted therapy is preferred because the adverse effects of targeted therapy are relatively more controllable. The LAUNCH trial, a randomized phase III study (LAUNCH trial) conducted in China, demonstrated a relative increase in median OS and PFS by 54.8% and 65.6%, respectively, when TACE-LEN was used as a first-line treatment option for patients with advanced HCC compared to lenvatinib monotherapy. Thus, the results of the LAUNCH trial brought new hope to patients with advanced HCC. However, the huge medical costs of TACE are a serious obstacle to its further expansion, thus necessitating a cost-effectiveness analysis of TACE-LEN. The results of our analysis showed that TACE-LEN was a cost-effective treatment option as the first-line therapy for advanced HCC compared with lenvatinib, at a WTP threshold of $38,201/QALY. The probability sensitivity analysis showed a 96.8% probability of cost-effectiveness, and the results of the subgroup analysis also support this cost-effectiveness finding. In addition, the participation rate of residents’ health insurance has now reached 96.8% in China. In addition, the participation rate of residents’ medical insurance in China has currently reached 96.8%. To our knowledge, this is the first cost-effectiveness analysis of TACE-LEN.

The reimbursement ratio for medical expenses incurred by medical insurance patients in tertiary hospitals is approximately 70%, with a higher percentage in primary healthcare institutions (Qin et al., 2023). Therefore, the actual probability of TACE-LEN being cost-effective may be higher for medical insurance patients. It is important to note that robotic surgery is increasingly being utilized in the treatment of HCC. It enhances surgical precision, reduces invasiveness, and assists surgeons in accessing hard-to-reach areas while minimizing blood loss and promoting faster recovery (Di Luca et al., 2020; Zhu et al., 2023). This holds particular benefits for patients with TACE-LEN treatment. In addition, the collapsibility of the inferior vena cava, a major conduit for deoxygenated blood returning to the heart, can be evaluated using subcostal and trans-hepatic ultrasound imaging. This assessment modality exhibits the potential for assessing the fluid status of patients with advanced HCC, warranting further investigation in this area (Sanfilippo et al., 2023; Zawadka et al., 2023).

Up till now, only two pharmacoeconomic studies had compared TACE with other treatment modalities for advanced HCC (Chen et al., 2018; Zhang et al., 2022), both of which used TACE alone as the therapeutic modality. The study by Zhang et al. (2022) showed that compared to hepatic arterial infusion chemotherapy, TACE was not cost-effective as a first-line treatment option for large unresectable HCC. Similarly, Chen et al. (2018) reported that TACE was not cost-effective as a first-line treatment option for advanced HCC compared with full-dose or dose-adjusted sorafenib. The possible reasons for the inconsistency of these results with our study are that treatment with TACE alone usually makes complete tumor necrosis difficult and then creates a secondary hypoxic environment within the residual lesion. Hypoxia stimulates the expression of angiogenic factors such as VEGF and FGF, which induces tumor progression, recurrence, and metastasis (Sergio et al., 2008; Shim et al., 2008; Chen et al., 2022), and subsequently, HCC patients show insensitivity or resistance to TACE leading to poor prognosis (Kudo et al., 2014; Zhong et al., 2021).

The comparison object selection is an important concern while performing cost-effectiveness analysis using the Markov model. According to the guidelines for the diagnosis and treatment of primary HCC, in addition to lenvatinib, atezolizumab plus bevacizumab and sorafenib are also the first-line treatment for advanced HCC. Currently, we lack robust head-to-head trial data to adequately compare the cost-effectiveness of TACE-LEN and various first-line therapies for advanced HCC. A study by Finn et al. (2020) found better OS and PFS outcomes with atezolizumab plus bevacizumab than with sorafenib for the treatment of unresectable HCC. However, the two China-based economic studies found atezolizumab plus bevacizumab to not be cost-effective compared to sorafenib (Hou and Wu, 2020; Wen et al., 2021). In addition, a study by Cai et al. (2020) found that from the perspective of the Chinese health delivery system, lenvatinib was a cost-effective targeted agent for unresectable HCC when compared to sorafenib. Therefore, we believe that it is reasonable to select lenvatinib as a comparator for the economic analysis of TACE-LEN.

The huge difference in economic development between different provinces in China is a problem that cannot be ignored, and many provinces’ GDP *per capita* does not reach the national average, which makes the results of our economic analysis bring some challenges in informing the actual medical work (Li et al., 2021; Zhang et al., 2022). Data from the National Bureau of Statistics show in 2022 that Gansu’s GDP *per capita* ($6,684), the lowest in China, is only 52.4% of the national average (Compiled by National Bureau of Statistics of China, 2023). Therefore, we need to explore the probability that TACE-LEN is cost-effective by continuously lowering the WTP threshold to accommodate the needs of provinces with lower levels of economic development. When we lowered the WTP threshold to 79.8% of the original preset level, i.e., $30482.13/QALY, the probability of TACE-LEN being cost-effective was 50%. That is, when three times the *per capita* GDP of a province is less than $30482.13, TACE-LEN is not cost-effective in that province. These results provide some economic reference for the selection of first-line treatment options for advanced HCC in low-income provinces in China.

Our analysis also has several limitations. First, the cost of weight-decreased treatment was as per the local medical price in Fujian, as it is not nationally consistent. Although this may lead to some bias, sensitivity analysis showed that it did not affect the model results. Second, due to the lack of long-term survival data, we used a log-Logistic survival model to infer survival tails beyond the follow-up time frame, which may not accurately reflect real-world conditions. We intend to update our cost-effectiveness analysis when long-term survival data are reported. Third, to simplify the model, we assumed that all patients received a 12 mg daily dose of lenvatinib, which may not correspond to our treatment reality. Nevertheless, the sensitivity analysis showed that the parameters associated with lenvatinib had little effect on the model results. Fourth, when patients experienced disease progression, we chose to put all patients on BSC due to the lack of relevant survival data for the enrolled patients, which may not accurately reflect current clinical practice. We will analyze this further when relevant treatment costs and survival data for patients after progression are available. Fifth, because the LAUNCH trial failed to provide quality-of-life data, the utility values in the model were derived from NICE and published literature, which may have led to bias in our model results. Finally, we considered only grade 3 or higher adverse events with a probability of occurrence greater than 5% in the model. We assumed that low-probability adverse events would not change the conclusions of the study; sensitivity analyses also showed that the economic results were insensitive to parameters related to adverse reactions.

## 5 Conclusion

Our study found that compared to lenvatinib, TACE-LEN is a cost-effective option as a first-line treatment for advanced HCC from a Chinese healthcare system perspective, but not so in low-income provinces in China. Although TACE-LEN is not currently included as a first-line treatment option as per Chinese HCC guidelines, our findings provide an important economic rationale for Chinese guideline developers, including those in low-income areas, to decide on the suitability of TACE-LEN as a first-line treatment option for advanced HCC.

## Data Availability

The original contributions presented in the study are included in the article/Supplementary Material, further inquiries can be directed to the corresponding authors.
