# Supplementary material for: Cost-effectiveness analysis of transarterial chemoembolization combined with lenvatinib as the first-line treatment for advanced hepatocellular carcinoma
Source: Front Pharmacol. 2023 Sep 7;14:1219694. doi: 10.3389/fphar.2023.1219694 (PMC10512705; doi:10.3389/fphar.2023.1219694)
Supplement: Supplementary file 1 [file Table1.docx]

Supplementary Material

**Cost-Effectiveness Analysis of Transarterial Chemoembolization Combined with Lenvatinib as the First-Line Treatment for Advanced Hepatocellular Carcinoma**

**1. Supplementary Table A. CHEERS 2022 Checklist.**

**2. Supplementary Table B. Comparison of survival models.**

**3. Supplementary Figure A. Results of all survival curves fit the lenvatinib group.**

**4. Supplementary Figure B: Result of probabilistic sensitivity analyses (scatter plot).**

**1.** **Supplementary Table A. CHEERS 2022 Checklist.**

| **Topic** | **No.** | **Item** | **Reported** |
| --- | --- | --- | --- |
| **Title** |  |  |  |
|  | 1 | Identify the study as an economic evaluation and specify the interventions being compared. | Yes |
| **Abstract** |  |  |  |
|  | 2 | Provide a structured summary that highlights context, key methods, results, and alternative analyses. | Yes |
| **Introduction** |  |  |  |
| **Background and objectives** | 3 | Give the context for the study, the study question, and its practical relevance for decision making in policy or practice. | Yes |
| **Methods** |  |  |  |
| **Health economic analysis plan** | 4 | Indicate whether a health economic analysis plan was developed and where available. | Not applicable |
| **Study population** | 5 | Describe characteristics of the study population (such as age range, demographics, socioeconomic, or clinical characteristics). | Yes |
| **Setting and location** | 6 | Provide relevant contextual information that may influence findings. | Yes |
| **Comparators** | 7 | Describe the interventions or strategies being compared and why chosen. | Yes |
| **Perspective** | 8 | State the perspective(s) adopted by the study and why chosen. | Yes |
| **Time horizon** | 9 | State the time horizon for the study and why appropriate. | Yes |
| **Discount rate** | 10 | Report the discount rate(s) and reason chosen. | Yes |
| **Selection of outcomes** | 11 | Describe what outcomes were used as the measure(s) of benefit(s) and harm(s). | Yes |
| **Measurement of outcomes** | 12 | Describe how outcomes used to capture benefit(s) and harm(s) were measured. | Yes |
| **Valuation of outcomes** | 13 | Describe the population and methods used to measure and value outcomes. | Yes |
| **Measurement and valuation of resources and costs** | 14 | Describe how costs were valued. | Yes |
| **Currency, price date, and conversion** | 15 | Report the dates of the estimated resource quantities and unit costs, plus the currency and year of conversion. | Yes |
| **Rationale and description of model** | 16 | If modelling is used, describe in detail and why used. Report if the model is publicly available and where it can be accessed. | Yes |
| **Analytics and assumptions** | 17 | Describe any methods for analysing or statistically transforming data, any extrapolation methods, and approaches for validating any model used. | Yes |
| **Characterising heterogeneity** | 18 | Describe any methods used for estimating how the results of the study vary for subgroups. | Yes |
| **Characterising distributional effects** | 19 | Describe how impacts are distributed across different individuals or adjustments made to reflect priority populations. | Yes |
| **Characterising uncertainty** | 20 | Describe methods to characterise any sources of uncertainty in the analysis. | Yes |
| **Approach to engagement with patients and others affected by the study** | 21 | Describe any approaches to engage patients or service recipients, the general public, communities, or stakeholders (such as clinicians or payers) in the design of the study. | Not applicable |
| **Results** |  |  |  |
| **Study parameters** | 22 | Report all analytic inputs (such as values, ranges, references) including uncertainty or distributional assumptions. | Yes |
| **Summary of main results** | 23 | Report the mean values for the main categories of costs and outcomes of interest and summarise them in the most appropriate overall measure. | Yes |
| **Effect of uncertainty** | 24 | Describe how uncertainty about analytic judgments, inputs, or projections affect findings. Report the effect of choice of discount rate and time horizon, if applicable. | Yes |
| **Effect of engagement with patients and others affected by the study** | 25 | Report on any difference patient/service recipient, general public, community, or stakeholder involvement made to the approach or findings of the study | Not applicable |
| **Discussion** |  |  |  |
| **Study findings, limitations, generalisability, and current knowledge** | 26 | Report key findings, limitations, ethical or equity considerations not captured, and how these could affect patients, policy, or practice. | Yes |
| **Other relevant information** |  |  |  |
| **Source of funding** | 27 | Describe how the study was funded and any role of the funder in the identification, design, conduct, and reporting of the analysis | Yes |
| **Conflicts of interest** | 28 | Report authors conflicts of interest according to journal or International Committee of Medical Journal Editors requirements. | Yes |

**2.** **Supplementary table B. Comparison of survival models**

| **Distributions** | **OS** | | **PFS** | |
| --- | --- | --- | --- | --- |
|  | **AIC** | **BIC** | **AIC** | **BIC** |
| Exponential | 805.532 | 808.656 | 900.362 | 903.486 |
| Gamma | 739.941 | 746.189 | 830.845 | 837.093 |
| Generalised F | 728.213 | 740.709 | 823.764 | 836.260 |
| Generalised Gamma | 735.570 | 744.942 | 824.670 | 834.042 |
| Gompertz | 780.596 | 786.844 | 881.010 | 887.258 |
| Weibull AF | 751.095 | 757.343 | 845.449 | 851.697 |
| Weibull PH | 751.095 | 757.343 | 845.449 | 851.697 |
| log-Logistic | 730.391 | 736.639 | 820.073 | 826.320 |
| log-Normal | 733.718 | 739.966 | 822.672 | 828.920 |

AIC, Akaike information criterion; BIC, Bayesian information criterion; OS, overall survival; PFS, progression-free survival.

**3.** **Supplementary Figure A. Results of all survival curves fit the lenvatinib group.**

**
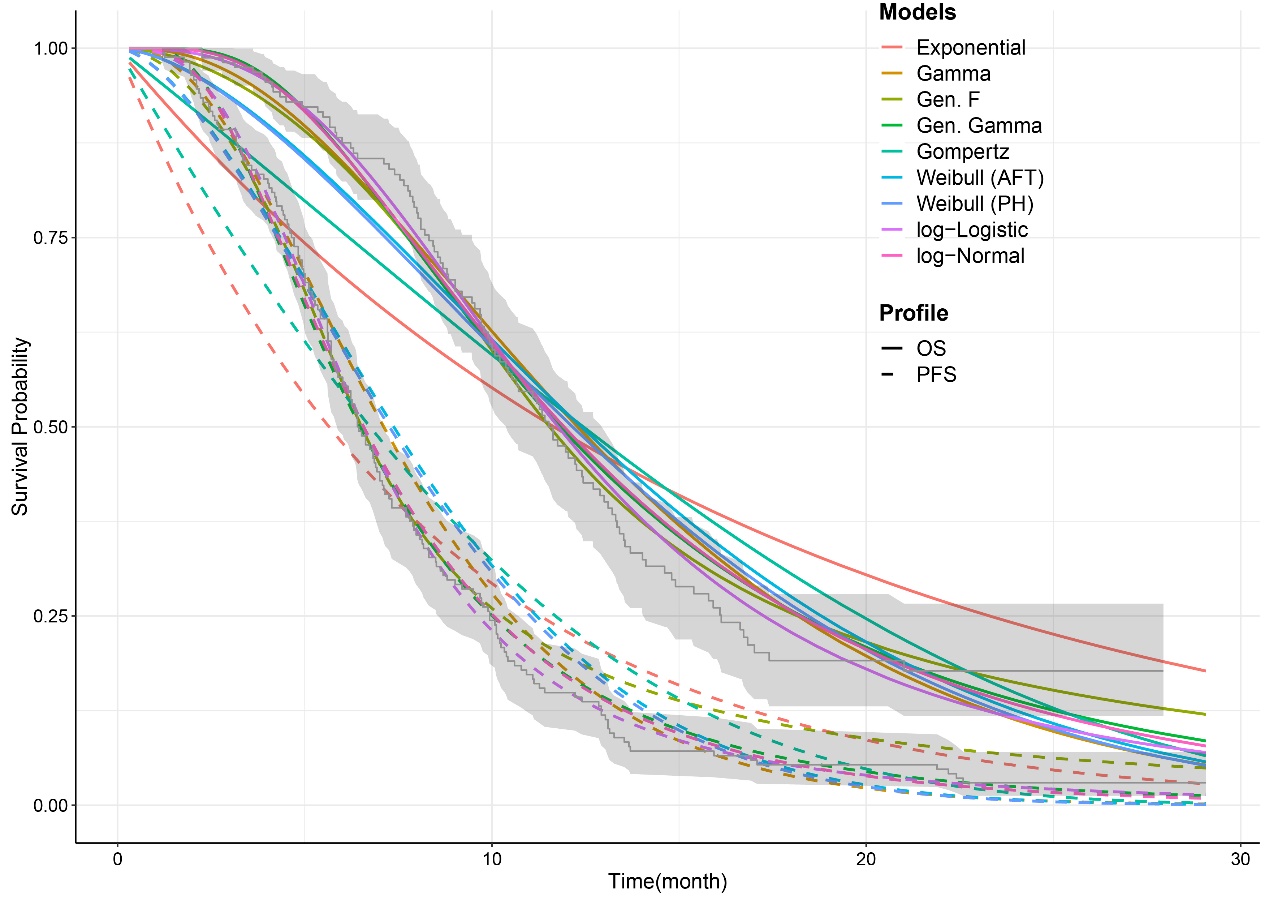
**

OS: Overall survival; PFS, progression-free survival.

**4. Supplementary Figure B: Result of probabilistic sensitivity analysis (scatter plot).**


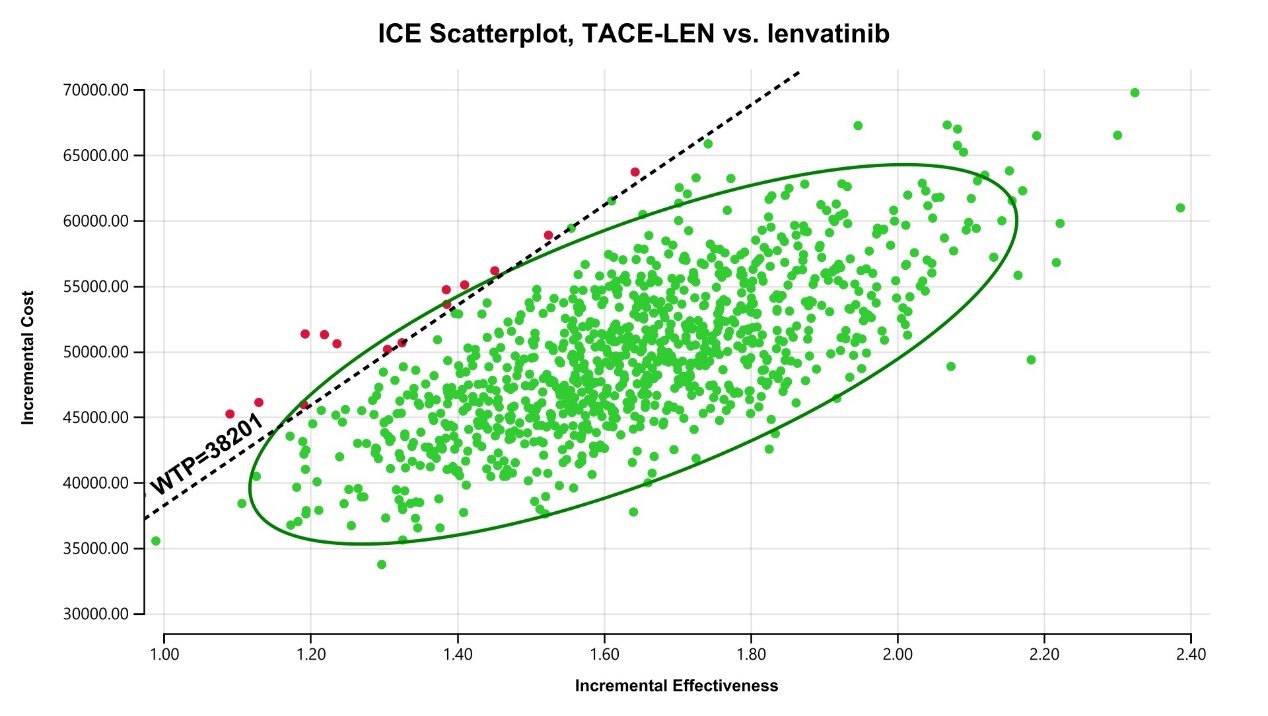


A probabilistic scatter plot of the ICER between the TACE-LEN and lenvatinib groups. Each point means the ICER for 1 simulation. Ellipses are used to indicate 95% confidence intervals. Points that lie below the ICER threshold represent cost-effective simulations. TACE-LEN, transarterial chemoembolization in combination with lenvatinib; WTP, willingness-to-pay.
